# Implication of dorsostriatal D3 receptors in motivational processes: a potential target for neuropsychiatric symptoms in Parkinson’s disease

**DOI:** 10.1038/srep41589

**Published:** 2017-01-30

**Authors:** Mathieu Favier, Carole Carcenac, Guillaume Drui, Yvan Vachez, Sabrina Boulet, Marc Savasta, Sebastien Carnicella

**Affiliations:** 1 Inserm, U1216, F- 38000 Grenoble, France; 2 Univ. Grenoble Alpes, Grenoble Institut des Neurosciences, GIN, F-38000 Grenoble, France; 3 Centre Hospitalier Universitaire de Grenoble, BP217, Grenoble F-38043, France

## Abstract

Beyond classical motor symptoms, motivational and affective deficits are frequently observed in Parkinson’s disease (PD), dramatically impairing the quality of life of patients. Using bilateral 6-hydroxydopamine (6-OHDA) lesions of the substantia nigra *pars compacta* (SNc) in rats, we have been able to reproduce these neuropsychiatric/non-motor impairments. The present study describes how bilateral 6-OHDA SNc lesions affect the function of the main striatal dopaminergic (DA) receptor subtypes. Autoradiography was used to measure the levels of striatal DA receptors, and operant sucrose self-administration and neuropharmacological approaches were combined to investigate the causal implication of specific DA receptors subtypes in the motivational deficits induced by a dorsostriatal DA denervation. We found that D3 receptors (D_3_R) exclusively are down-regulated within the dorsal striatum of lesioned rats. We next showed that infusion of a D_3_R antagonist (SB-277011A) in non-lesioned animals specifically disrupts preparatory, but not consummatory behaviors. Our findings reveal an unexpected involvement of dorsostriatal D_3_R in motivational processes. They strongly suggest an implication of dorsostriatal D_3_R in the neuropsychiatric symptoms observed in PD, highlighting this receptor as a potential target for pharmacological treatment.

Beyond the characteristic motor symptoms linked to the degeneration of dopaminergic (DA) neurons in the substantia nigra *pars compacta* (SNc), Parkinson’s disease (PD) has recently been identified as a quintessential neuropsychiatric disorder^1^,^2^,^3^. Apathy is one of the most frequently reported non-motor features of the disease and therefore contributes, at least as much as the motor symptoms, to impair quality of life and to high morbidity in patients^1^,^4^,^5^. Apathy is operationally defined as a lack of motivation or a reduction in goal-directed behaviors^6^,^7^ resulting clinically in a deficit of self-initiated, voluntary and purposeful behavior. Apathy is also frequently associated with affective disorders such as anxiety and depression^1^,^5^. Schmidt *et al*. recently demonstrated that apathetic patients suffered from incapacity to translate expected reward into suitable efforts and actions, while perception of reward value remained unaffected^8^. This thereby indicates that apathy is specifically related to severe dysfunctions of motivational preparatory processes. In addition, apathetic symptoms in PD patients have been shown to occur particularly in early untreated PD patients or in conditions in which DA medications are highly reduced, while they can be greatly ameliorated by DA replacement therapies^2^,^3^. Apathy in PD thus appears to fluctuate with the DA state of the patient, suggesting a critical role of DA transmission in the pathophysiology of this neuropsychiatric syndrome^2^,^3^. Using a lesion-based approach with the infusion of 6-hydroxydopamine (6-OHDA) in distinct parts of the DA mesencephalon in rats, we have recently demonstrated that motivational deficits, reminiscent of apathetic symptoms in PD, may directly result from the loss of nigral DA neurons^9^, at least in part. We found that bilateral DA lesions of the SNc induced a profound decrease in operant performances for sucrose self-administration without alteration of the reinforcing/rewarding value of sucrose, suggesting a specific impact on the preparatory component of motivated behaviors, as observed in apathetic PD patients. Moreover, SNc DA lesions lead also to the development of anxiety- and depression-related behaviors^9^,^10^. Importantly, these behavioral deficits were corrected by DA drugs, notably D_2_/D_3_R agonists, known to alleviate apathetic and depressive symptoms in PD patients^9^,^10^,^11^ (see also for review^12^). Taken together, these data clearly indicated a critical role of nigrostriatal DA dysfunctions in the pathophysiology of non-motor disorders of PD, but the underlying mechanisms remain unknown.

The various functions of DA are mediated by different DA receptor subtypes and depend on their neuronal and brain localizations^13^. Moreover, expression and function of these DA receptors can be strongly affected by DA denervation, with potential pathophysiological implications in PD^14^,^15^. Here, we therefore set out to determine whether dysfunction of a specific DA receptor subtype-mediated neurotransmission would contribute to the motivational deficits induced by the SNc DA lesion. Using semi-quantitative autoradiographic analysis, we evaluated modifications in the expression of D_1_, D_2_ and D_3_ receptors (D_1_R, D_2_R and D_3_R) in our model and found a selective decrease in D_3_R levels within the dorsal striatum of SNc-lesioned rats. Moreover, dorsostriatal infusion of a specific D_3_R antagonist (SB-277011A) in non-lesioned rats mimicked the behavioral deficits induced by DA lesion, suggesting that the motivational deficits observed in SNc-lesioned rats are causally related to the functional downregulation of dorsostriatal D_3_R.

## Results

### 6-OHDA-lesioned rats exhibit a selective decrease of D_3_R expression within the dorsal striatum

**Figure 1:**
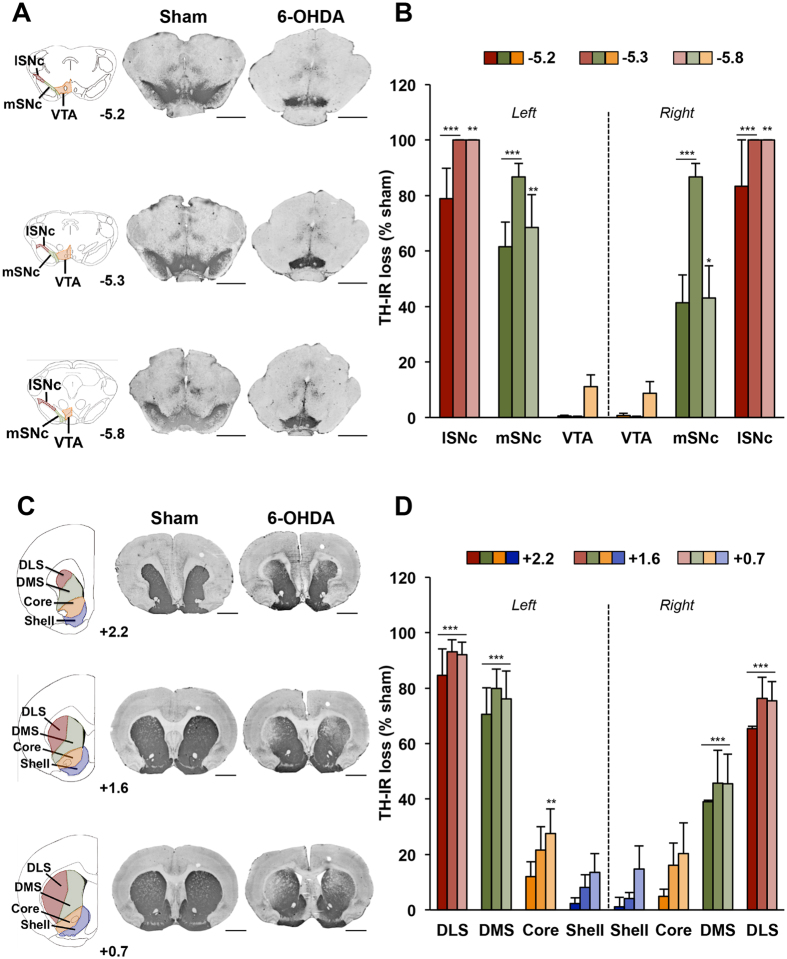
Mesencephalic and striatal DA denervation induced by bilateral 6-OHDA lesions of the SNc. (**A**–**C**) Photographs of coronal sections stained for TH at mesencephalic (**A**) and striatal (**C**) levels and corresponding diagrams showing the masks used for the quantification of DA denervation in the different subregions analyzed. AP levels are indicated in mm from bregma. Scale: 2 mm. 6-OHDA: 6-hydroxydopamine. (**B**–**D**), Quantification of the loss of TH staining at mesencephalic (**B**) and striatal (**D**) levels, expressed as a percentage of the mean value obtained for sham-operated animals. Two-way ANOVAs and *post-hoc* analyses with the method of contrasts were used. *p < 0.05, **p < 0.01, ***p < 0.001, sham *vs* 6-OHDA (sham, n = 8; 6-OHDA, n = 7). AP: Anteroposterior; DLS: dorsolateral striatum; DMS: dorsomedial striatum; l-mSNc: lateral-medial substantia nigra *pars compacta*; VTA: ventral tegmental area.

**Figure 2:**
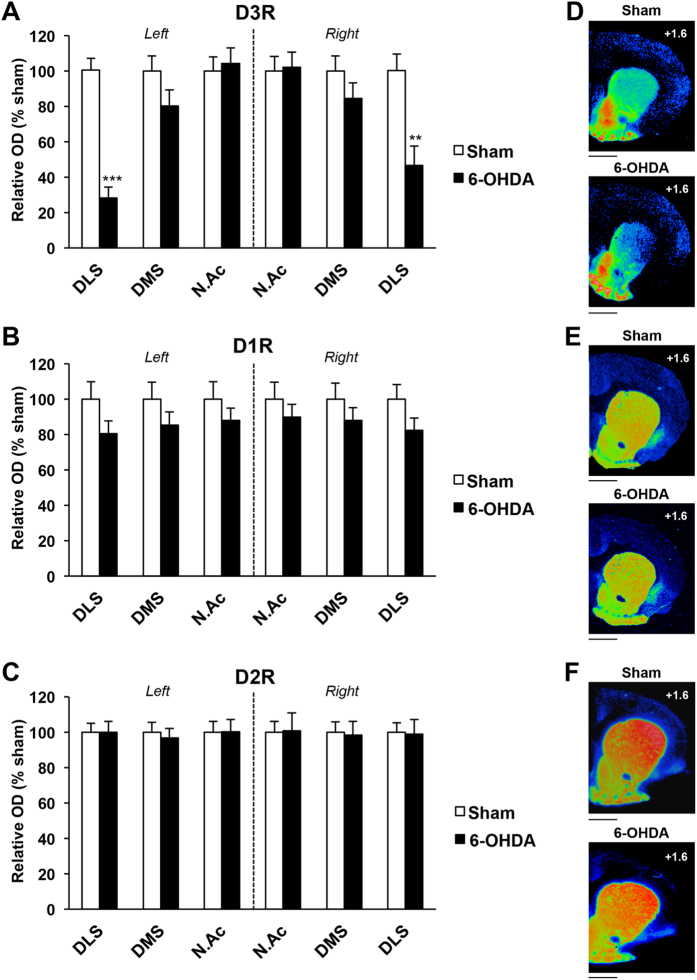
Bilateral 6-OHDA SNc lesion induces a selective decrease of D_3_R expression in dorsolateral striatum. (**A**–**C**), Mean ± SEM optical density (expressed as arbitrary units) of D_**3**_R, D_**1**_R and D_**2**_R receptor binding density at striatal level, as measured by semi-quantitative autoradiography in sham and 6-OHDA lesioned rats. SNc lesions induced a marked decrease of D_3_R binding, specifically within the dorsolateral striatum. Two-way ANOVAs and *post-hoc* analyses with the method of contrasts were used. **p < 0.01, ***p < 0.001, sham (n = 8) *vs* 6-OHDA (n = 7). DLS: dorsolateral striatum; DMS: dorsomedial striatum; NAc: nucleus accumbens. (**D**–**F**) Photographs of autoradiograms obtained at striatal level for sham and 6-OHDA–lesioned rats, colorized using Autoradio V4.03 Software. D, D_3_R; E, D_1_R; F, D_2_R. AP levels are indicated from bregma. Scale: 1mm. AP: Anteroposterior.

We first tested whether partial and bilateral DA denervation of the nigrostriatal system is accompanied by modifications of the expression of D_1_R, D_2_R and D_3_R, as evaluated by semi-quantitative autoradiography within different parts of the striatum. As previously shown^9^,^11^, loss of nigral DA neurons (Fig. 1A and B) induced by posterior and bilateral infusion of 6-OHDA led to a 70–80% bilateral loss of tyrosine hydroxylase immunoreactivity restricted to the dorsal striatum (Fig. 1C and D, dorsomedial striatum, DMS + dorsolateral striatum, DLS: 73.7 ± 6.1%), and predominantly in its more lateral part. The NAc was relatively unaffected (Core + Shell: 12.5 ± 5.3%) (Fig. 1C and D; lesion x structure interaction, whatever the striatal level considered: F_3,104_ > 31.64, ps < 0.001). This DA denervation was associated with a selective reduction of D_3_R radiolabeling within the DLS (as well as with a marginal decrease in the DMS: p = 0.13 and 0.23 for the left and the right side, respectively) (Fig. 2A and Table 1), whereas D_1_R and D_2_R radiolabeling were not modified (Fig. 2B and C and Table 1). This was supported by a three-way ANOVA which showed a significant lesion x structure x receptor interaction (F_4,117_ = 2.82, p < 0.05). These data therefore suggest that partial and bilateral DA denervation of the nigrostriatal system induce a specific downregulation of dorsostriatal D_3_R.

### Infusion of a selective D_3_R antagonist into the dorsal striatum reduces operant sucrose self-administration

**Figure 3:**
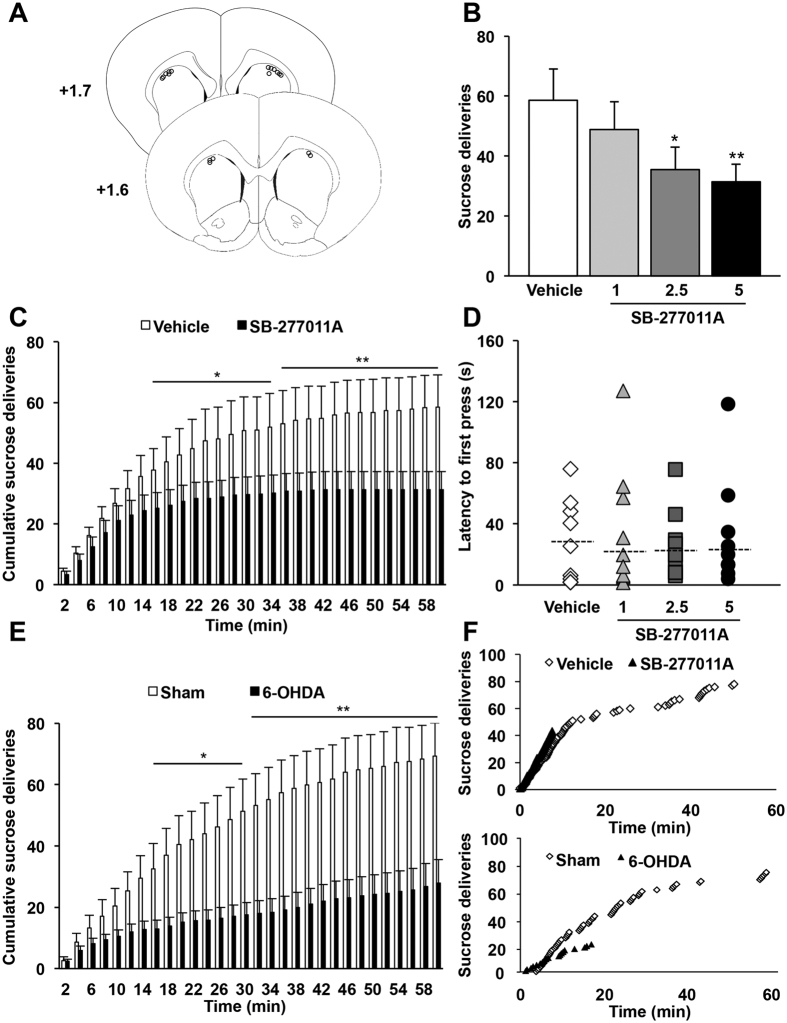
Intracerebral infusion into the dorsal striatum of a selective D_3_R antagonist decreases operant sucrose self-administration. (**A**) Diagrams showing the locations of each individual guide cannula for the animals included in behavioral experiments (n = 9). The circles represent the tips of the microinjectors, visualized on coronal sections counterstained with cresyl violet. AP levels are indicated in mm from bregma. (**B**) Mean ± SEM number of sucrose deliveries over 60-minute sessions. Dorsostriatal infusion of the D_3_R antagonist SB-277011A dose-dependently decreased instrumental performance for sucrose self-administration. One-way repeated ANOVA and *post-hoc* analyses with Student-Newman-Keuls test were used. *p < 0.05, **p < 0.01, vehicle *vs* SB-277011A (n = 9 per group). (**C**) Mean ± SEM number of cumulative sucrose deliveries within 2-minute bins over 60 minutes sessions. SB-277011A administration (5 μg.μl^−1^) specifically affected maintaining the instrumental response while no effect was seen during the first part of the procedure. Two-way repeated ANOVA and *post-hoc* analyses with the method of contrasts were used. *p < 0.05, **p < 0.01, vehicle *vs* SB-277011A (n = 9 per group). (**D**) Median latencies for the first active lever press (dotted bars) and individual values. SB-277011A administration did not modify the latency for the first active lever press (Friedman test). (**E**) Mean ± SEM number of cumulative sucrose deliveries within 2-minute bins over 60-minute operant self-administration sessions for sham *vs* 6-OHDA SNc-lesioned rats. Two-way ANOVAs and *post-hoc* analyses with the method of contrasts were used. *p < 0.05, **p < 0.01, sham (n = 7) *vs* 6-OHDA (n = 7). (**F**) Individual data showing similar profiles of early termination of instrumental activity observed after pharmacological inhibition of D_3_R in dorsal striatum (same rat before or after injection of vehicle *vs* SB-277011A, 5 μg.μl^−1^) or after SNc 6-OHDA lesion. Each microinjection experiment with vehicle *vs* SB-277011A was conducted by using a counterbalanced within-subject design. AP: Anteroposterior.

Because D_3_R have been proposed to regulate emotional and motivational functions^16^,^17^, we hypothesized that such downregulation of D_3_R in the dorsal striatum may contribute to the motivational deficit induced by 6-OHDA-lesion. Thus, we tested whether pharmacological blockade of the D_3_R within the dorsal striatum with the selective antagonist SB-277011A^18^,^19^ would reduce operant sucrose self-administration in non-lesioned rats, as observed after SNc DA lesion^9^. Dorsostriatal infusion of SB-277011A (Fig. 3A) induced a dose-dependent decrease in the number of sucrose deliveries over the 60 min session (Fig. 3B; main effect of treatment: F_3,24_ = 5.30, p < 0.01). By analyzing the cumulative number of reward deliveries (Fig. 3C) and latencies to the first press (Fig. 3D), we found that SB-277011A infusion, even at the highest dose did not affect the initiation phase, but specifically induced an early termination of instrumental activity (Fig. 3C, main effect of treatment and time, and significant interaction between both factors: F_s_ > 3.86, p_s_ < 0.05; Fig. 3D, Χ^2^ = 1.27; p = 0.74). Importantly, and although SNc DA lesions induced a greater decrease in operant sucrose self-administration, a similar profile was observed in SB-277011A-treated non-lesioned rats, with no modification of first press latencies^11^ and an early interruption of operant activity (Fig. 3E and F).

**Figure 4:**
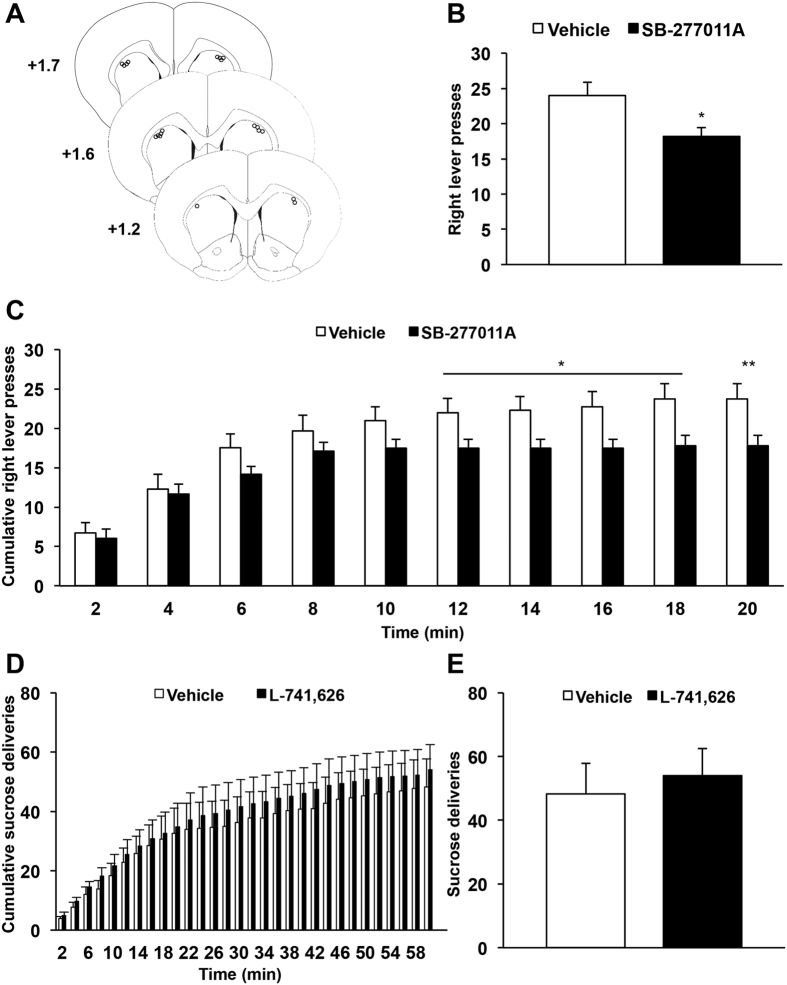
Dorsostriatal infusion of a selective D_3_R antagonist specifically affects preparatory components of motivated behavior whilst D_2_R antagonist does not modify operant sucrose self-administration. (**A**) Diagrams showing the locations of each individual guide cannula for the animals included in behavioral experiments (n = 10). The circles represent the tips of the microinjectors, visualized on coronal sections counterstained with cresyl violet. AP levels are indicated in mm from bregma. (**B**) Mean ± SEM number of right lever presses over 20-minute extinction seeking sessions. SB-277011A (5 μg.μl^−1^) administration induced a significant decrease in seeking behaviors during an extinction procedure (*t*-test). (**C**) Mean ± SEM number of cumulative right lever presses within 2 minutes bins over 20 minutes extinction seeking sessions. The effect of SB-277011A on operant sucrose self-administration was specifically observed during the maintenance but not the initiation of seeking behavior. Two-way repeated ANOVA and *post-hoc* analyses with method of contrasts test were used. *p < 0.05, **p < 0.01, vehicle *vs* SB-277011A (n = 10 per group). (**D**) Mean ± SEM number of cumulative sucrose deliveries within 2-minute bins over 60-minute operant self-administration sessions. (**E**) Mean ± SEM number of sucrose deliveries over 60-minute operant self-administration sessions. Dorsostriatal infusion of a D_2_R antagonist (L-741,626, 2.5 μg.μl^−1^) did not modify operant sucrose self-administration. Two-way repeated ANOVA and *post-hoc* analyses with the method of contrasts (cumulative number of sucrose deliveries) or *t*-test (total number of sucrose deliveries) were used, vehicle *vs* L-741,626 (n = 10 per group). Each microinjection experiment was conducted using a counterbalanced within-subject design. AP: Anteroposterior.

Moreover, although dorsostriatal infusion of D_3_R antagonist induced a deficit in operant activity, reward deliveries remained rapidly followed by reward port entries, with a pattern comparable to that observed in the vehicle condition (Supplemental Figs 1 and 2), indicating that consummatory behaviors were not affected by the blockade of D_3_R. By taking advantage of an extinction procedure to dissociate seeking from consummatory behaviors^20^, we then showed that infusion of SB-277011A into the dorsal striatum (Fig. 4A) produced a significant reduction in the number of lever presses (Fig. 4B), remarkably similar to the one observed during operant sucrose self-administration. Indeed, dorsostriatal infusion of the D_3_R antagonist did not affect initiation of seeking but induced an early termination of instrumental activity (Fig. 4C, marginal effect of treatment: F_1,72_ = 5.26, p = 0.051, but significant interaction between treatment and time: F_9,72_ = 2.20, p < 0.05). Taken together, these data strongly suggest that SB-277011A in the dorsal striatum impairs preparatory behaviors, as observed in SNc-lesioned rats^9^, by specifically affecting the maintenance of seeking behavior.

Finally, this effect is likely mediated by a selective blockade of D_3_R and not to the structurally related D_2_R, as infusing a specific D_2_R antagonist (L-741,626^21^) into the dorsal striatum did not alter operant sucrose self-administration (Fig. 4D, no effect of treatment: F_s_ < 0.91, p_s_ > 0.36 and no time x treatment interaction: F_s_ < 0.66, p_s_ > 0.71; and Fig. 4E, p = 0.39).

### Infusion of a selective D_3_R antagonist into the dorsal striatum does not induce either sensorimotor deficits nor anhedonia

**Figure 5:**
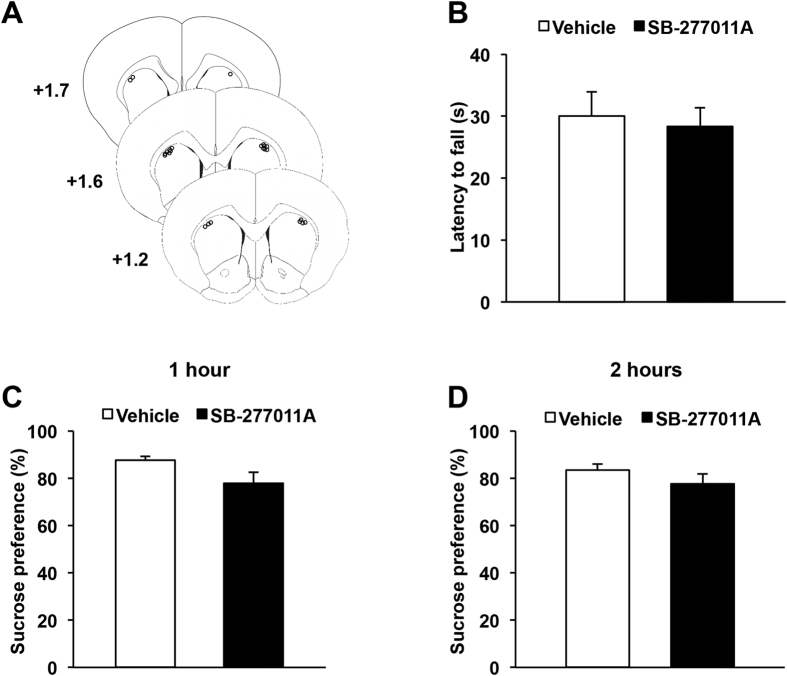
Dorsostriatal infusion of a selective D_3_R antagonist neither affects sensorimotor coordination nor consummatory behaviors. (**A**) Diagrams showing the locations of each individual guide cannula of animals included in behavioral experiments (n = 12). The circles represent the tips of the microinjectors, visualized on coronal sections counterstained with cresyl violet. AP levels are indicated in mm from bregma. (**B**) Mean ± SEM latencies to fall from an accelerating rotarod. This latency was not altered by dorsostriatal infusion of SB-277011A (*t*-test). (**C**,**D**) Mean ± SEM preference for sucrose over water in a 2 hours free-access session. (**C**) Preference measured after 1 hour. (**D**), Preference measured after 2 hours. Dorsostriatal infusion of SB-277011A (5 μg.μl^−1^) did not modify sucrose preference, neither after 1 hour nor at the end of the 2 hours session. (*t*-test). Vehicle *vs* SB-277011A (n = 12 per group). Each microinjection experiment was conducted by using a counterbalanced within-subject design. AP: Anteroposterior.

Although the absence of effect of the D_3_R antagonist on initiation of operant behaviors (Figs 3C and D and 4C) suggests a preservation of motor functions, we verified that infusion of SB-277011A into the dorsal striatum does not alter the sensorimotor coordination of the animals (Fig. 5A). As shown in Fig. 5B, latencies to fall from an accelerating rotarod were not modified by the infusion of the highest dose of SB-277011A used in the instrumental procedure (p = 0.55), thereby confirming that the decrease in operant sucrose self-administration induced by D_3_R inhibition did not result from a motor deficit.

We have previously demonstrated that the deficit in operant performance in SNc-lesioned rat was not related to a decrease in sensitivity to the motivational properties of sucrose^9^. We therefore tested the effect of the administration of SB-277011A into the dorsal striatum on preference for sucrose over water in a 2 hour two-bottle choice procedure. As shown in Fig. 5C and D, preference for the sucrose solution was not altered by dorsostriatal infusion of the highest dose of the D_3_R antagonist used in the instrumental procedure, either after 1 hour or 2 hours of access (p = 0.35 and p = 0.22, respectively). Consistently, neither sucrose nor water intake were modified by the drug treatment, at the two time-points (data not shown, p_s_ > 0.22). Taken with the aforementioned results, it indicates that SB-277011A in the dorsal striatum specifically impairs preparatory, but not consummatory components of motivated behavior, as observed in SNc-lesioned rats^9^.

### Infusion of a selective D_3_R antagonist into the NAc does not reduce operant sucrose self-administration

**Figure 6:**
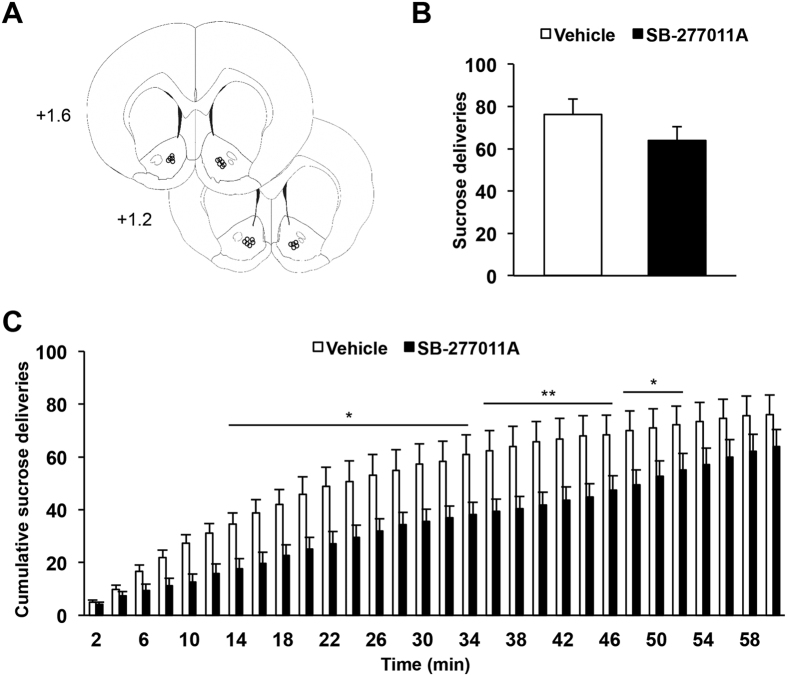
Intracerebral infusion of a selective D_3_R antagonist into the nucleus accumbens does not reduce operant sucrose self-administration. (**A**) Diagrams showing the locations of each individual guide cannula of animals included in behavioral experiments (n = 11). The circles represent the tips of the microinjectors, visualized on coronal sections counterstained with cresyl violet. AP levels are indicated in mm from bregma. (**B**) Mean ± SEM number of sucrose deliveries over 60 minutes sessions. (**C**) Mean ± SEM number of cumulative sucrose deliveries within 2 minutes bins over 60 minutes operant self-administration sessions. Intra-accumbal infusion of SB-277011A (5 μg.μl^−1^) did not modify instrumental performance for sucrose self-administration over the entire duration of the test, but instrumental performance of the animals were temporarily decreased in the middle period of the test. Two-way repeated ANOVA and *post-hoc* analyses with the method of contrasts (cumulative number of sucrose deliveries) or *t*-test (total number of sucrose deliveries) were used. *p < 0.05, **p < 0.01, vehicle *vs* SB-277011A (n = 11 per group). Each microinjection experiment was conducted by using a counterbalanced within-subject design. AP: Anteroposterior. Favier *et al*., Table 1.

In order to verify the anatomical specificity of the effects of the D_3_R antagonist, SB-277011A was infused into the NAc (Fig. 6A), the neighboring striatal region critically involved in reward-related and motivated behaviors^22^,^23^. In contrast to its administration into the dorsal striatum, intra-accumbal infusion of the highest dose of SB-277011A did not significantly alter operant sucrose self-administration over the 60 minutes of the test session (Fig. 6B, p = 0.08). Analysis of the temporal distribution of sucrose deliveries however, revealed a transient and discrete deficit in operant performances for rats administrated with SB-277011A into the NAc (Fig. 6C, main effect of treatment: F_1,319_ = 6.27, p = 0.03, and time x treatment interaction: F_29,319_ = 80.86, p < 0.001), that sharply contrasts with the early-termination effect observed after infusion into the dorsal striatum. Taken together, these results suggest that the strong deficit in operant sucrose self-administration induced by the blockade of D_3_R is specific for the dorsal striatum.

## Discussion

Using a validated DA lesion-based model of PD-related motivational deficits in rats^9^,^11^,^24^, we demonstrate that partial and selective loss of DA innervation in the dorsal striatum is accompanied by downregulation of D_3_R within this area. Furthermore, pharmacological blocking of the D_3_R within the dorsal striatum, but not in the NAc, phenocopies the deficits in preparatory behaviors observed in this model, suggesting that the downregulation of dorsostriatal D_3_R plays a critical role in the motivational deficits detected following this selective DA lesion. Taken together, our results point towards an unsuspected role of dorsostriatal D_3_R in motivational processes and could represent a potential pharmacological target to treat PD-related neuropsychiatric symptoms.

D_3_R is the only DA receptor subtype for which the expression was strongly affected by the partial denervation of the DA nigrostriatal system. In physiological conditions, D_3_R expression is high in the NAc shell, intermediate in the NAc core and lower, but detectable, in the dorsal striatum^25^,^26^. Because of this low expression, investigation of the possible modification of D_3_R expression in the dorsal striatum following PD-related DA lesions remain challenging and have yielded conflicting results. For instance, previous studies reported a decrease or no change in dorsostriatal D_3_R expression in DA-depleted rats, depending on the method of detection and analysis (e.g protein or mRNA^27^,^28^,^29^). Here, using an adapted protocol to detect changes of expression in brain areas where D_3_R expression is low^30^,^31^, we demonstrate that D_3_R is specifically downregulated in the DLS after bilateral SNc 6 OHDA-lesion. In the striatum, D_3_R can mediate the action of DA as a post-synaptic receptor or exert an inhibitory effect on DA release as a putative pre-synaptic receptor^13^. However, D_3_R autoradiographic binding and mRNA levels are highly colocalized in the striatum, thereby suggesting that these localizations primarily correspond to dendrites or soma of striatal neurons rather than to long axon terminals from distant neurons^32^ and the decreased D_3_R expression in the present study is associated with a phenotype related to a loss of dopaminergic function (observed in SNc-lesioned rats), reproduced by the pharmacological blockade of dorsostriatal D_3_R. We therefore hypothesize that the loss of D_3_R was due, at least in part, to changes of expression in post-synaptic neurons.

Conversely, D_1_R and D_2_R expression levels were not modified after bilateral SNc 6-OHDA-lesion. There is a lack of general consensus for D_1_R, but our results are consistent with literature reporting no or marginal modification of expression depending on the PD animal models used^33^,^34^,^35^. For D_2_R, converging data, even in humans, suggested that this receptor subtype is over-expressed at the striatal level, but only after total or subtotal dorsostriatal DA denervation^15^,^34^,^35^. Indeed, a direct relationship between D_2_R overexpression and appearance of PD motor symptoms has been suggested^36^,^37^. Therefore, a lack of modification of D_2_R levels, as observed here, is consistent with the preservation of the motor function described in our experimental model^9^,^11^. Finally, since D_2_R are located pre- and post-synaptically, we cannot exclude that the absence of radiolabeling changes for this receptor subtype results from a combination of a concomitant pre-synaptic decrease and a post-synaptic increase in D_2_R expression^33^,^38^.

The implication of the D_3_R subtype in PD non-motor deficits has rarely been investigated in animals^39^. Because modulation of the D_3_R function influences motivated behaviors^16^,^17^, it seems reasonable to hypothesize that downregulation of this receptor, restricted to the dorsal striatum, may participate in the motivational deficits induced by the DA SNc lesion. Indeed, our data show that selective blockade of D_3_R within the dorsal striatum in non-lesioned rats impairs operant sucrose self-administration, and specifically the maintenance of the operant response, without affecting the rewarding properties of the reinforcer, as observed in lesioned rats^9^. In this study, we used the D_3_R antagonist SB-277011A since it is considered to be one of the most selective compound for D_3_R^18^,^19^, contrary to others D_3_R blockers displaying more mixed specificity for D_2_R and D_3_R^40^. In addition, we confirmed that the behavioral effects of SB-277011A were not mediated by D_2_R, as infusion of a selective D_2_R receptor, L-741,626^21^ in the dorsal striatum, had no effect on the operant performances of the animals. Although both blockade of D_3_R in the dorsal striatum and SNc DA lesions impair the maintenance of operant behaviors, the effect on operant performance of the latter^9^,^11^ is stronger than of the former. Moreover, the curves showing the pattern of operant activity across time appear similar, but not identical. In addition to the decrease of D_3_R expression revealed by autoradiography in the present study, SNc-lesioned rats also exhibited a dramatic reduction (−70%) of extracellular DA levels in the dorsal striatum^11^. These cumulative DA dysfunctions in 6-OHDA rats may account for these differences, indicating that pharmacological blockade of dorsostriatal D_3_R may reproduce only a part of the deficit induced by the SNc lesion. Taken together, these data clearly emphasize a critical, while probably non-exclusive, implication of dorsostriatal D_3_R in the pathophysiology of motivational deficits in PD patients related to apathy.

With the notable exception of experimental studies on drugs of abuse and L-Dopa-induced dyskinesia^26^,^27^,^28^, the role of dorsostriatal D_3_R in motivational processes remains poorly investigated because of its low basal level of expression within this area. We show that infusion of the selective D_3_R antagonist in the dorsal striatum specifically alters the maintenance, but not the initiation of operant sucrose self-administration. This effect is highly reminiscent of the “extinction-like” effect observed after systemic^41^ and, interestingly, dorsostriatal but not accumbal, intracerebral infusions^42^ of broad-spectrum DA antagonists at moderate doses. However, and contrary to the initial postulate of Wise^43^, a decrease in the hedonic/rewarding effects of sucrose cannot account for this result. Indeed, the effect of the D_3_R antagonist on maintenance of operant behaviors was observed even in absence of the reward, and neither consummatory behaviors nor preference for the sucrose solution in a two-bottle choice procedure were affected by the dorsostriatal infusion of the D_3_R antagonist. Therefore, these data clearly indicate that blocking or reducing D_3_R transmission within the dorsal striatum induces a specific deficit in goal-oriented behavior. According to the concept of incentive salience developed by Berridge and collaborators^22^,^44^, DA signaling is necessary for transforming the “neutral” perception of a conditioned stimulus, or a goal object at a distance into an attractive incentive capable of eliciting appetitive or instrumental behaviors towards it (wanting). During motivational tasks, incentive salience assignment to reward-related stimuli and actions are maintained or strengthened by the presentation of the reinforcer (i.e, a correct prediction has been made). It is proposed that DA mediates this “reboosting” effect so that the reinforcer and associated cues remains “wanted” at later occasions^22^,^44^. We can thus hypothesize that blockade of dorsostriatal D3R may induce a specific impairment of “reboosting” processes, accounting for the early termination in instrumental activity reported in the present study. Accordingly, Howe *et al*. recently showed that prolonged tonic DA signals, or “ramps”, detected by fast-scan cyclic voltammetry in the dorsal striatum could provide sustained motivational drive needed to maintain instrumental behaviors^45^, while this region, especially in its lateral part, has also been shown to mediate the properties of reward-related stimuli to stimulate operant responding^46^. Our findings suggest that, despite a limited level of expression, dorsostriatal D3R may have a critical functional implication in these DA-mediated processes.

Finally, in contrast to the dorsal striatum, infusion of a D_3_R antagonist into the NAc did not affect the maintenance of instrumental behaviors, but rather induced a discrete decrease in the rate of responses for sucrose. The effects related to D_3_R blockade in the NAc are consistent with the literature since in operant tasks with low ratio requirement, as in the FR1 procedure used here, only slowing in the rate of responding and increased tendency to pause are observed after blockade of DA receptors or DA depletion in the NAc^23^,^47^. This differential implication of accumbal and dorsostriatal D_3_R in instrumental behaviors and motivational processes deserves further investigation, notably by relying on cue-driven reward-seeking procedures^48^,^49^.

In conclusion, these data strengthen the role of the nigrostriatal DA system in motivational processes by uncovering a new function of dorsostriatal D_3_R and emphasize a critical role of dorsostriatal D_3_R neurotransmission in the pathophysiology of PD-related neuropsychiatric disorders. In view of recent clinical and preclinical data suggesting that stimulation of D_3_R may have important beneficial effects in depression and apathy in PD^2^,^24^,^50^, the present study clearly highlights D_3_R as a relevant target for the treatment of some major, invalidating, non-motor symptoms of PD.

## Methods

See Supplementary Methods and Materials for details regarding surgeries concerning 6-OHDA lesions and guide cannulae implantation, neuroanatomical controls of the implantations, brain processing for immunohistochemistry and autoradiographic experiments, as well as data and statistical analyses.

### Animals

Experiments were performed on male Sprague Dawley rats (Janvier, Le Genest-Saint-Isle, France) weighing 220 g (6 weeks old) at the time of surgery (dopaminergic lesion or guide cannulae implantation). Animals were housed four per cage, until the second week after surgery and then transferred to individual cages until the end of the study, under standard laboratory conditions (12 h light/dark cycle, with lights on at 7 a.m.), with food and water supplied *ad libitum*. All experimental protocols complied with the European Union 2010 Animal Welfare Act and the new French directive 2010/63, and were approved by the French national ethics committee no. 004.

### Tyrosine hydroxylase (TH) immunohistochemistry and quantification of DA denervation

#### TH immunohistochemistry

Immunostaining was carried out as previously described^9^,^11^,^24^, except that sections collected on microscope slides were first air-dried and post-fixed with 4% paraformaldehyde (PFA) for 10 min, and then washed in PBS. Briefly, sections from the mesencephalon and the striatum were incubated with an anti-TH antibody (mouse monoclonal MAB5280, Millipore, France, 1:2500) and then with a biotinylated goat anti-mouse IgG antibody (BA-9200, Vector Laboratories, Burlingame, CA, USA; 1:500). Immunoreactivity was visualized with avidin-peroxidase conjugate (Vectastain ABC Elite, Vector Laboratories Burlingame, CA, USA).

#### Quantification and analysis

TH immunoreactivity (TH-IR) was analyzed with the ICS FrameWork computerized image analysis system (Calopix, 2.9.2 version, TRIBVN, Châtillon, France) coupled to a light microscope (Nikon, Eclipse 80i) and a Pike F-421C camera (ALLIED Vision Technologies, Stadtroda, Germany) for digitalization, in the VTA, the SNc (−5.2 to −5.8 mm from bregma), the dorsal striatum and the NAc (+2.2 to +0.7 mm from bregma), taking into account the topography of DA innervation^51^,^52^. Masks from these different subregions were drawn with the computer analysis system to ensure that appropriate comparisons were made between homologous anatomical regions. Optical densities (OD) were measured for each striatal and mesencephalic subregion, and the mean OD was calculated with ICS FrameWork software (TRIBVN, 2.9.2 version, Châtillon, France). OD were expressed as percentages relative to the mean optical density values obtained from the homologous regions of sham-operated animals. Mean bilateral TH-IR loss within the dorsal striatum (i.e, DMS + DLS) had to be between 50% and 85%, with a TH-IR loss in the NAc that did not exceed 30%, for inclusion in the analysis, as previously determined^9^,^11^.

### DA receptors autoradiography

#### *D*_*1*_ *R*

Tissue sections were air-dried and then pre-incubated for 30 min with 50 mM Tris-HCl pH 7.4, 120 mM NaCl, 5 mM KCl, 2 mM CaCl_2_, 1 mM MgCl_2_ (buffer A). Sections were incubated for 1 h at room temperature (RT) in buffer A supplemented with 2 nM [^3^H]-SCH23390 (Perkin Elmer, Courtaboeuf, France) (buffer B). Nonspecific binding was determined by incubation with buffer B supplemented by cis-flupenthixol. Sections were rinsed in cold water for 30 sec, twice 10 min in cold 50 mM Tris-HCl, and finally twice 15 sec in cold water. Sections were then dried and placed against phosphor screens (BAS-IP-TR-2025 E, Dominique Dutscher, France) for 5 days in light-proof X-ray cassettes. ^3^H microscales (RPA 506, 3-109.4 nCi.mg^−1^, Amersham Biosciences, France) were also placed against phosphor screens for comparison. Screens were finally analyzed using a phosphoimager (Fujifilm BAS-5000, France).

#### *D*_*2*_ *R*

Tissue sections were air-dried, pre-incubated 3 × 5 min with buffer A, and then incubated for 1 h at RT in buffer A supplemented with 0.2 nM [^125^I]-Iodosulpride (Perkin Elmer, Courtaboeuf, France) (buffer C). Nonspecific binding was determined by incubation with buffer C supplemented with 10 μM apomorphine. Sections were rinsed 4 × 5 min in cold 50 mM, Tris-HCl, 15 sec in cold water, then air-dried and placed against X-ray films (Biomax MR, Kodak, Sigma-Aldrich, Saint Quentin-Fallavier, France) for 4 days in light-proof X-ray cassettes. The films were developed by immersion (10 sec) in Kodak GBX Developer at RT, fixed using Kodak GBX Fixer (3 min), rinsed in deionized water (Kodak processing chemicals for autoradiographic films, Sigma-Aldrich, Saint Quentin-Fallavier, France) and dried.

#### *D*_*3*_ *R*

Tissue sections were air-dried and pre-incubated 3 × 4 min with buffer containing 50 mM HEPES pH 7.4 and 1 mM EDTA, freshly supplemented with 50 mg.L^−1^ ascorbic acid, 1 g.L^−1^ bovine serum albumin (BSA), 100 μM guanosine5′-triphosphate (GTP), and 25 μM 1,3-di(2-tolyl)guanidine (DTG) in methanol (buffer D). Sections were incubated for 1 h at RT in buffer D supplemented with [^125^I]-7OH-PIPAT (Perkin Elmer, Courtaboeuf, France) (buffer E). Nonspecific binding was determined by incubation with buffer E supplemented with 10 μM dopamine. Sections were rinsed 4 × 15 min in cooled 50 mM HEPES, 5 sec in cold water, then dried and placed against X-ray films (Biomax MR, Kodak) for 8 days in light-proof X-ray cassettes. The films were developped by immersion (20 sec) in Kodak GBX Developer at RT, fixed using Kodak GBX Fixer (3 min), rinsed in deionized water (Kodak processing chemicals for autoradiographic films, Sigma-Aldrich, Saint Quentin-Fallavier, France) and dried.

#### Quantification of DA receptor binding

Image files obtained with phosphoimager (D_1_R) converted to appropriate file format using Adobe Photoshop, and autoradiograms obtained from X-ray films (D_2_R and D_3_R) were digitized (Capture NX2 Nikon software). OD values (expressed in arbitrary units) and colorized images were derived with Autoradio V4.03 software (SAMBA Technologies, Meylan, France). For each rat, three stained sections of the same AP level were used for quantification and the triplicate OD values obtained for each region of interest (ROI) were averaged. For each ROI, the OD value obtained for nonspecific binding was subtracted from the corresponding OD value obtained for total binding.

### Drug microinjection procedure

Before injection, obturators were gently removed from the guide cannulae and microinjection cannulae (33 gauges, Plastic One, USA) were inserted bilaterally to extend 0.5 mm below the end of the guides. Then, 1 μl drug or vehicle was infused at a flow rate of 0.5 μl.min^−1^ 20. After a 2 min post-infusion period to allow diffusion of the solutions, microinjectors were removed and the obturators were reinserted. Finally, rats were returned to home cages for 5 min before behavioral testing. Each microinjection experiment was conducted by using a counterbalanced within-subject design, with at least 3 days before 2 infusions allowing full recovery and stabilization of behavioral performances. SB-277011A (Santa Cruz Biotechnology, Heidelberg, Germany) was dissolved in 20% dimethyl sulfoxyde (DMSO) in sterile 0.9% NaCl^53^ to concentrations of 1, 2.5 and 5 μg.μl^−1^, based on a pilot study and the literature^54^,^55^. SB-277011A is selective for D_3_R^18^,^19^, contrary to others D_3_R blockers displaying mixed specificity for D_2_R and D_3_R^40^. L-741,626 (Sigma-Aldrich, Saint Quentin-Fallavier, France) was dissolved in 5% DMSO and Cremophor sterile 0.9% NaCl, to a concentration of 2.5 μg.μl^−1^, as previously described^55^,^56^.

### Behavioral procedures

#### Operant sucrose self-administration

Rats were first trained to self-administer a 2.5% sucrose solution in operant chambers (Med Associates, St. Albans, VT, USA), as previously described^9^,^11^,^24^, under a fixed ratio 1 schedule of reinforcement (FR1), with an active, reinforced, lever, for which presses resulted in the delivery of 0.2 ml of the sucrose solution, and an inactive, non-reinforced, lever, for 1 h. Because the number of presses on the inactive lever was extremely low after acquisition of the self-administration procedure (<10 presses), and the activity on this lever was not affected by any of the experimental treatments, this measure was excluded from the figures and the analysis for better clarity. Once their performance was stabilized (less than 20% performance variation over three consecutive sessions), rats were bilaterally implanted with guide cannulae. After at least 3 days post-surgery recovery, rats were again trained to self-administer sucrose to obtain a stable baseline of performances before starting the microinjection procedure.

#### Extinction seeking test

After stabilization of operant performances, rats experienced a single 20 min session of extinction, in which responses on the active lever were counted but no sucrose was delivered^20^.

#### Accelerating rotarod

Animals were trained to remain on a rotarod (Harvard Apparatus, Holliston, MA, USA). The rotation speed of the rod was gradually increased, from 4 to 40 r.p.m, at a rate of 1 r.p.m. every 8 s for 5 min maximum^9^. After 3 days training, rats were microinjected as described above, and latency to fall from the rod was recorded three times for each rat.

#### Evaluation of sucrose preference

Rats were given 2-h concurrent access in their home cage to two graduated 250 ml plastic bottles with modified sipper to prevent spillage (Techniplast, Lyon, France), for 3 days. One of these bottles contained tap water, whereas the other contained 2.5% sucrose in tap water. Bottles were weighed after 1 h and at the end of the 2 h test period, with the position of the bottles (left or right) alternated to control for side preference. The volumes of sucrose solution and water consumed were averaged to determine sucrose, water and total fluid intake (ml.kg^−1^), as well as preference for sucrose over water (sucrose intake/total intake, expressed as a percentage). After 3 days training, animals showed stable consumption (less than 20% variation over two consecutive days) and were microinjected as described above.

## Additional Information

**How to cite this article**: Favier, M. *et al*. Implication of dorsostriatal D3 receptors in motivational processes: a potential target for neuropsychiatric symptoms in Parkinson’s disease. *Sci. Rep.* **7**, 41589; doi: 10.1038/srep41589 (2017).

**Publisher's note:** Springer Nature remains neutral with regard to jurisdictional claims in published maps and institutional affiliations.

## Supplementary Material

Supplementary Information

## Figures and Tables

**Table 1:** Effect of SNc lesion on the expression of striatal DA receptors subtypes.

Structure	Left side		Right side	
	Sham	6-OHDA	Sham	6-OHDA
D_3_R				
DLS	7.3 (±0.5)	2.06*** (±0.4)	7.4 (±0.7)	3.4** (±0.8)
DMS	11.9 (±1)	9.5 (±1.1)	12.9 (±1.1)	10.9 (±1.1)
N.Ac	23.8 (±1.2)	24.8 (±1.2)	23.7 (±2)	24.2 (±2.3)
D_1_R				
DLS	36.6 (±3.6)	29.5 (±2.6)	36.5 (±3.4)	30.1 (±2.6)
DMS	38.4 (±3.7)	32.7 (±2.7)	38.4 (±3.4)	33.7 (±2.8)
N.Ac	37.1 (±3.7)	32.6 (±2.6)	37.6 (±3.2)	33.8 (±2.6)
D_2_R				
DLS	85.9 (±4.4)	85.8 (±5.4)	81 (±4.4)	80.1 (±6.6)
DMS	74.7 (±4.1)	72.2 (±4.1)	73.9 (±4.3)	72.6 (±5.9)
N.Ac	53.4 (±3.2)	53.6 (±3.6)	53.7 (±3.3)	54.2 (±5.4)

Modifications of expression of D_3_R, D_1_R, and D_2_R at striatal level induced by a bilateral 6-OHDA SNc lesion. Values of relative optical densities (arbitrary units) are expressed as a mean ± SEM. Two-way ANOVAs and *post-hoc* analyses with the method of contrasts were used.

**p < 0.01, ***p < 0.001, sham (n = 8) *vs* 6-OHDA (n = 7). DLS: dorsolateral striatum; DMS: dorsomedial striatum; NAc: nucleus accumbens.
