# Supplementary material for: Implication of dorsostriatal D3 receptors in motivational processes: a potential target for neuropsychiatric symptoms in Parkinson’s disease
Source: Sci Rep. 2017 Jan 30;7:41589. doi: 10.1038/srep41589 (PMC5278505; doi:10.1038/srep41589)
Supplement: Supplementary Information [file srep41589-s1.pdf]

Implication of dorsostriatal D3 receptors in motivational processes: a potential target for neuropsychiatric symptoms in Parkinson's disease

Authors: Mathieu Favier[1,2], Carole Carcenac[1,2], Guillaume Drui[1,2], Yvan Vachez[1,2], Sabrina Boulet[1,2], Marc Savasta[1,2,3]* and Sebastien Carnicella[1,2]*

Supplementary information

Supplementary Methods

Bilateral 6-hydroxydopamine (6-OHDA) lesions

As previously described[1], rats were anesthetized with a mixture of xylazine (15 mg.kg^{-1} intraperitoneally, i.p.) and ketamine (100 mg.kg^{-1}, i.p.) and treated with desipramine hydrochloride (25 mg.kg^{-1} subcutaneously; Sigma-Aldrich, St Quentin-Fallavier, France) 30 min before 6-OHDA injection, to protect noradrenergic neurons[2]. Rats were secured in a Kopf stereotaxic apparatus (Phymep, Paris, France) and 6 µg 6-OHDA (Sigma-Aldrich) dissolved in 2.3 µl sterile 0.9% NaCl ,or 2.3 µl sterile 0.9% NaCl (sham conditions), were injected bilaterally (flow rate of 0.5 µl.min^{-1}) at the following coordinates relative to bregma[3]: AP, -5.4 mm; ML, ±1.8 mm; DV, -8.1 mm. The relatively posterior placement of the injectors is determinant for sparing part of the DA ascending fibers, to obtain only partial (<80%) DA denervation of the dorsal striatum, thus circumventing severe alterations of motor function[4]. After each injection, the cannula was left in position for 5 min to allow the injected solution to be absorbed and to minimize the spread of the toxin along the needle tract. After recovery from anesthesia, animals were returned to the animal facility for three weeks, to allow the 6-OHDA lesion to develop and stabilize[2] before the beginning of autoradiographic experiments. A subset of animals (around 20%) received supplementation with a high-caloric liquid diet and palatable food for 1 to 2 weeks, as transient starvation states may occur during the development of the DA SNc lesions[1].

Implantation of guide cannulae

Rats were anaesthetized by a mixture of xylazine (15 $mg.kg^{-1}$ i.p.) and ketamine (100 $mg.kg^{-1}$, i.p.) and implanted with bilateral guide cannulae (26 gauge, Plastic One, Roanoke, USA) aimed at one of two brain region at the following coordinates relative to bregma[3] dorsal striatum: AP, +1.6 mm; ML, ±3.4 mm; DV, -0.5 mm or nucleus accumbens (NAc): AP, +1.6 mm; ML, ±1.5 mm; DV, -4.5 mm. We mainly targeted the anterolateral part of the dorsal striatum since we previously showed that the loss of TH in this area correlated with the development of motivational deficits[4]. Moreover, autoradiography showed that D3R expression was preferentially down-regulated in this area in SNc-lesioned rats (see Figure 2). Guide cannulae were lowered into place and attached to the skull by small stainless steel screws and dental acrylic. Obturators (33 gauge; Plastic One) were inserted to prevent obstruction.

Brain processing for immunohistochemistry and autoradiographic experiments

Animals were anaesthetized by chloral hydrate and perfused intracardially with 0.9% saline. Brains were quickly removed, frozen in cooled (-40°C) isopentane, then stored at -30°C. Serial coronal sections (14 µm-thick and 30 µm-thick for autoradiography and immunohistochemistry, respectively) were cut with a cryostat (HM 500, Microm, Francheville, France), collected on microscope slides and stored at -30°C.

Histological control of guide cannulae implantation

All animals were perfused intracardially with 4% PFA. Brains were frozen in cooled (-40°C) isopentane and stored at -30°C. Locations of cannulae were verified in 30 µm cryostat-cut coronal sections, stained with cresyl violet.

Data and statistical analyses

Data were expressed as mean ± SEM and were analyzed by *t*-tests or one-, two- or three-way ANOVAs, with or without repeated measures, depending on the experimental design.

Post-hoc analyses were carried out with Student-Newman-Keuls test or the method of contrasts when indicated. Only latencies to the first sucrose delivery were represented with the median and individual values, as they were not normally distributed[4]. Analyses were therefore conducted using the Friedman repeated-measures ANOVA on ranks when indicated.

References

1 Drui, G. *et al.* Loss of dopaminergic nigrostriatal neurons accounts for the motivational and affective deficits in Parkinson's disease. *Mol Psychiatry* **19**, 358-367 (2014).

2 Schwarting, R. K. & Huston, J. P. Unilateral 6-hydroxydopamine lesions of meso-striatal dopamine neurons and their physiological sequelae. *Prog Neurobiol* **49**, 215-266 (1996).

3 Paxinos, G. & Watson, C. *The rat brain in stereotaxic coordinates*. fourth edn, (Elsevier Academic Press, 1998).

4 Favier, M. *et al.* Pramipexole reverses Parkinson's disease-related motivational deficits in rats. *Mov Disord* **29**, 912-920 (2014).

Favier et al., Suppl. Fig.1

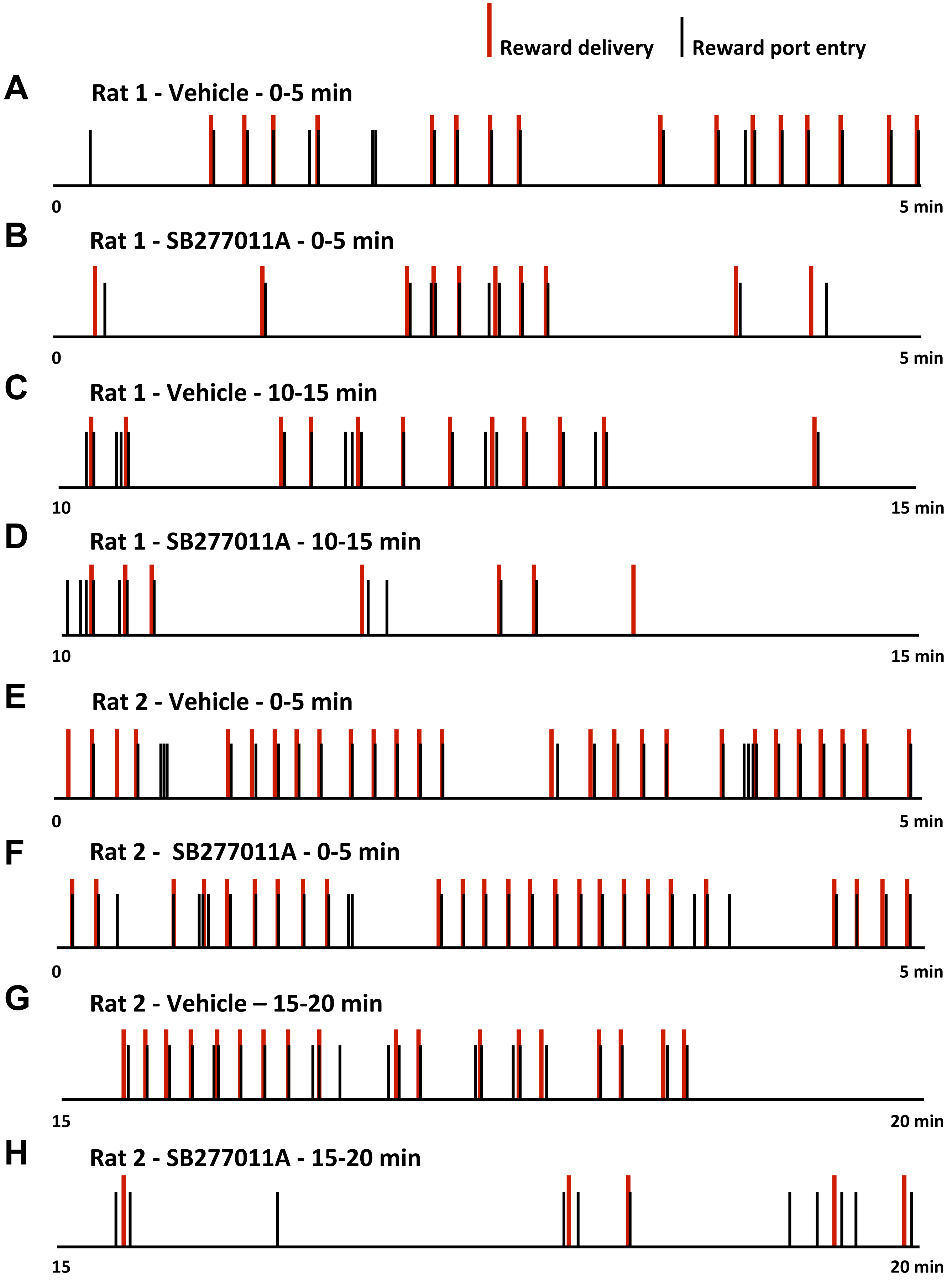

Favier et al., Suppl. Fig.2

A

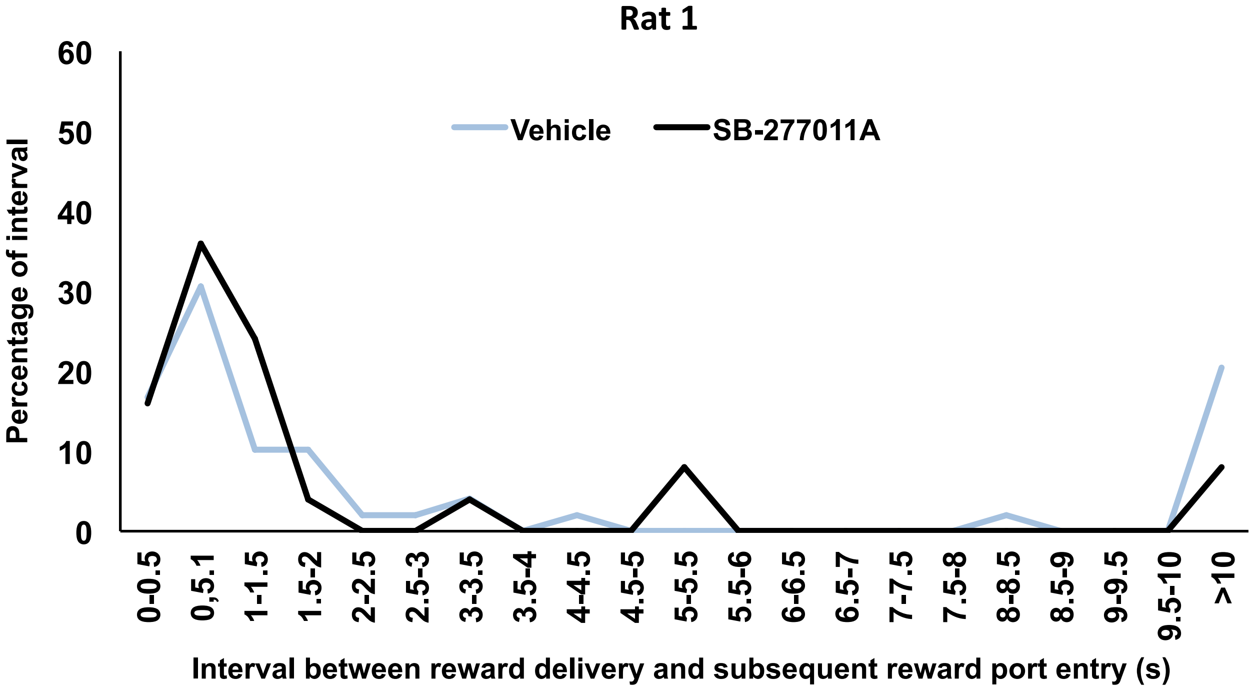

B

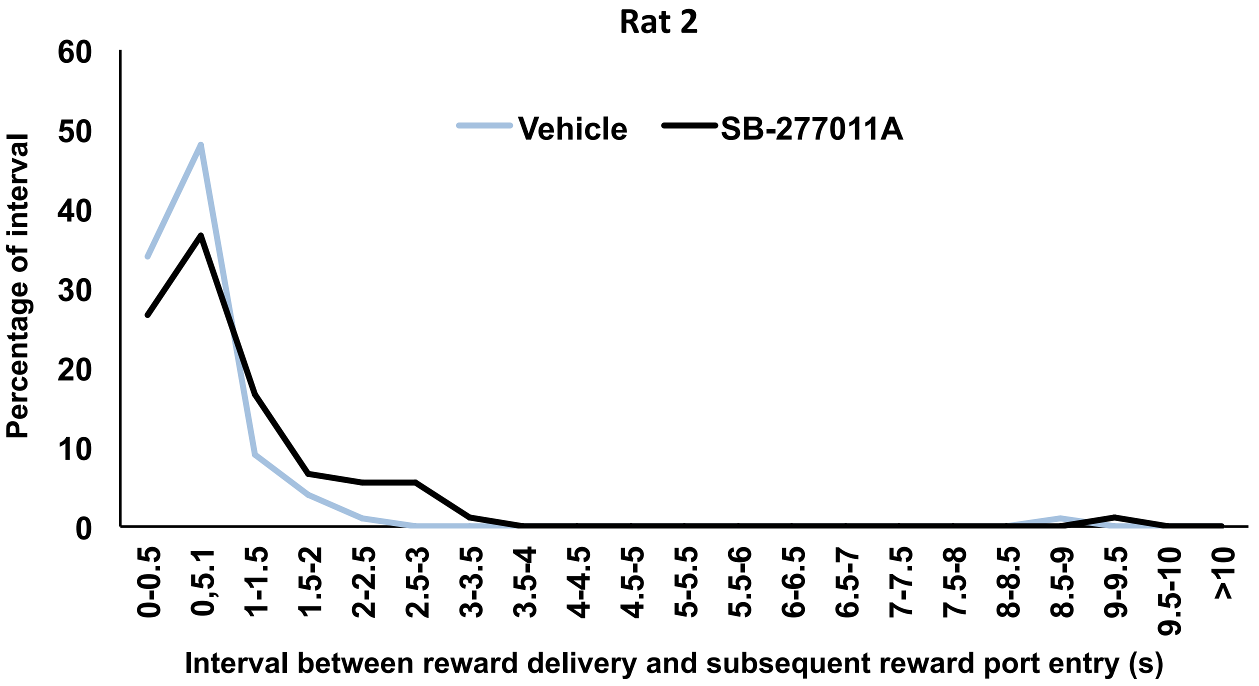

Supplemental Figure 1. Dorsostriatal infusion of a selective D_3R antagonist does not modify the distribution of reward port entries activity during sucrose self-administration procedure. A-H, Illustrations for two representative rats of the temporal distribution of reward deliveries (red bars) and reward port entries (black bars) during selected periods of 5 minutes of the operant session. A-D, Rat 1, pattern of events during the period 0 to 5 minutes, after vehicle (A) *vs* SB-277011A (B) infusion and during the period 10 to 15 minutes, after vehicle (C) *vs* SB-277011A (D) infusion. E-H, Rat 2, pattern of events during the period 0 to 5 minutes, after vehicle (E) *vs* SB-277011A (F) infusion and during the period 15 to 20 minutes, after vehicle (G) *vs* SB-277011A (H) infusion. Dorsostriatal infusion of D_3R antagonist induced a deficit in operant activity, reflected by a decrease in the number of reward deliveries. However, even in a SB-277011A condition when the operant activity was low, reward deliveries were followed by reward port entries, with a pattern comparable to that observed with vehicle alone.

Supplemental Figure 2. Dorsostriatal infusion of a selective D_3R antagonist does not modify the distribution of intervals between reward deliveries and subsequent reward port entries during sucrose self-administration procedure. A-B, Distribution of intervals for two representative rats over 60-minute sessions expressed as percentages, after vehicle (blue line) *vs* SB-277011A (black line) infusion. A, Rat 1. B, Rat 2. The vast majority of reward deliveries were rapidly followed by reward port entries, and this was not affected by the pharmacological inhibition of dorsotriatal D_3R.
